# Homœopathy and Thomsonism

**Published:** 1849-07

**Authors:** 


*Homœopathy and Thomsonism.*

One good effect sometimes resulting from the prevalence of medical delusions is, that persons who once become infatuated, and learn by experience the error of their ways, afterwards are proof against similar deceptions, and remain firm friends of legitimate medicine. A few years ago, Thomsonism was the rage in this region, and not a few respectable and intelligent individuals were drawn into the vortex. Most of them have long since repented of their folly, and are able to laugh at the ridiculous position in which some of their acquaintances are placed, who are carried away with the now fashionable humbug of infinitesimal quantities, the hair of the same dog, etc. We were somewhat amused, the other day, at the remark of one of our patients, who had once been a rabid Thomsonian. Said he :—"The difference between Homœopathy and Thomsonism, seems something like that between *petty* and *grand* larceny!" The idea intended to be conveyed, appeared to be, that while, in a moral aspect, they are the same in character, the Homœopathic system of deception seems to lack a kind of dignity, which the mind is apt to attach to a bold and extensive design upon the property or lives of others.

